# A Two-Component System Regulates Hemin Acquisition in *Porphyromonas gingivalis*

**DOI:** 10.1371/journal.pone.0073351

**Published:** 2013-09-05

**Authors:** Jodie C. Scott, Brian A. Klein, Ana Duran-Pinedo, Linden Hu, Margaret J. Duncan

**Affiliations:** 1 Department of Microbiology, The Forsyth Institute, Cambridge, Massachusetts, United States of America; 2 Department of Molecular Biology and Microbiology, Tufts University Sackler School of Biomedical Sciences, Boston, Massachusetts, United States of America; 3 Division of Geographic Medicine and Infectious Disease, Tufts Medical Center, Boston, Massachusetts, United States of America; Centre National de la Recherche Scientifique, Aix-Marseille Université, France

## Abstract

*Porphyromonas gingivalis* is a Gram-negative oral anaerobe associated with infection of the periodontia. The organism has a small number of two-component signal transduction systems, and after comparing genome sequences of strains W83 and ATCC 33277 we discovered that the latter was mutant in histidine kinase (PGN_0752), while the cognate response regulator (PGN_0753) remained intact. Microarray-based transcriptional profiling and ChIP-seq assays were carried out with an ATCC 33277 transconjugant containing the functional histidine kinase from strain W83 (PG0719). The data showed that the regulon of this signal transduction system contained genes that were involved in hemin acquisition, including gingipains, at least three transport systems, as well as being self-regulated. Direct regulation by the response regulator was confirmed by electrophoretic mobility shift assays. In addition, the system appears to be activated by hemin and the regulator acts as both an activator and repressor.

## Introduction

*Porphyromonas gingivalis,* a black-pigmented Gram-negative oral anaerobe, is a member of the oral microbiota. Although it is found in subgingival plaque of periodontally healthy individuals, this opportunistic pathogen is present in higher numbers in subjects with chronic periodontitis. *P.gingivalis* cells must adjust their physiology in order to survive the multiple challenges of the infectious process such as host defense products and metabolites produced by other microorganisms in the gingival biofilm. The ability to respond to such environmental changes is in part regulated by two component systems (TCS) that comprise a sensor histidine kinase (HK) and a response regulator (RR) [1]. Triggered by an environmental cue the HK autophosphorylates a conserved histidine within its sensor domain. The high-energy phosphate is then transferred to a conserved aspartate within the receiver domain of the cognate RR. The activated RR then binds to the promoter of a target gene to either induce or repress gene expression.

The genomes of *P.gingivalis* ATCC 33277, the type strain, and W83, a clinical isolate, contain four HK/RR pairs, one orphan HK, two orphan RR, and one HK-RR chimeric protein (http://ncbi.nlm.nih.gov/Complete_Genomes/SignalCensus.html,11; http://cmr.tigr.org/tigr-scripts/CMR/CmrHomePage.cgi) [2]. In strain W83, PG0719 (HK)-PG0720 (RR) [TIGR annotation] comprise a classical TCS, encoding 50 and 25 kDa proteins, respectively. Comparison of the genomes of strains W83 genome and TDC60 with that of ATCC 33277 revealed a 2.5 kb deletion in the latter in the region homologous to PG0719-0720. As a result, the HK of ATCC 33277 (PGN_0752) [NCBI annotation] appears to be rendered non-functional, and while the RR (PGN_0753) is structurally intact its expression could not be detected.

We used a combination of genetics, transcript expression profiling, and DNA-protein binding assays to identify components of the PGN_0753 regulon in *P.gingivalis* ATCC 33277. Among the candidate genes identified we focused on those involved in acquisition and transport of iron/hemin into cells, and named the system HaeSR (for **hae**min). Iron is an essential nutrient for survival of *P. gingivalis*, and the majority of iron found in the human body is stored in hemoglobin as heme (iron complexed with protoporphyrin IX). *P. gingivalis* has a number of mechanisms for sequestering heme from hemoglobin and other host proteins such as degradation and heme binding by gingipains, and outer membrane receptors with high affinity for heme that is subsequently transported into the cell [3]. We found that HaeSR directly regulated several uptake systems as well as expression of gingipain cysteine proteinases that degrade hemoglobin to release and bind heme. The present study indicates that the HaeSR system plays a significant role in regulating these processes.

## Materials and Methods

### Bacterial Strains, Plasmids, Media and Growth Conditions

Bacterial strains, plasmids, and PCR primers are listed in Table 1. The *P. gingivalis* strains W83 and ATCC 33277 used in this study were grown in trypticase soy broth (4% TSB), 2 µg/ml hemin, 1 µg/ml menadione, and also on plates which contained 5% sheep blood (BAP). The strains were grown at 37°C in an anaerobic chamber (Coy Laboratory Products) with 85% N_2_, 5% H_2_, and 10% CO_2_ for 3 to 5 days.

**Table 1:** Primers, strains, and plasmids used in this study.

Primers	Sequence 5′–3′ forward/reverse (amplicon size in bp)	Reference
**PG0719 cloning** ** in pT-COW**		
PG0719	GGATCCGATTCGGTTATAAACTCATAGAAAGAG/CYCGRGCGGCTGATCGAGC	This study
**PGN_0753 cloning** **in pET-22b^+^**		
PGN_0753-his	GGGCATATGATGAAAATCCTCATTATCG/TTTGGATCCGACGTACACCATCTCATCG	This study
**QRT-PCR**		
Pg 16SrRNA	ACAGTGGAGAGTTTCATGGTG/CATGGGTTCACCCCCCCTGTG	[36]
PG0719 W83	CTATGAGCTCAAAGTTTCCATGCCGACC/GCCGATCAGTCGATACAGTGGCCG	This study
PG0720 W83	GGGGCATCCCTTGGAGTTGAAGC/CATCGTTCATCTGCTTGCGCAGATTT	This study
PGN_0752 ATCC 33277	GTGAAGGCTCGTCGCGAAATGG/TTTTTTGTCATCCAGGCTCGCACTGAT	This study
PGN_0753 ATCC 33277	GGGGCATCCCTTGGAATTGAAGCG/TCGCCCCTGCATCGTTCATCTGCTTGCG	This study
*htrA* PGN_0687	ATCCTCGTGACAGCAAGCGTC/TCGAGTAGGGGGAGCGAGC	This study
*ihtA* PGN_0704	ACCGAACAAGGCAGATGCTTC/CCTTAGGCGTCATTCGTGTACCG	This study
*hmuY* PGN_0558	GGAAAATGGTGCCGTATTCTCC/ATTCCATCTGATGACCATCAGGACCC	This study
*ragA* PGN_0293	GGTATTTCCCGTGAGCCTTCTTCTTTCA/GGGTACCATTTAGCAATTCGCAATGGAC	This study
*hagA* PGN_1733	TGATGACGTGGCTGTTTCTGGTGA/TTGTACTGGCCGGGAGCTACATTT	This study
*rgpA* PGN_1970	TGGACAGGTTGTAAACTTTGCGCC/TTGCCTTGTTCCGAAGTTTCGCTC	This study
*bcp* PGN_1058	ACGATTATCGCGGACGAAAG/CAGCACGAAGTGTGCTATGA	This study
*hmuS* PGN_0558	GGCAAGACCGATGAGGATATTT/GATTGCCGAAGCCTATCAGTAG	This study
PGN_1343	ACTCCTTTCTATCCCTCCTACTC/TTTCTTCTGTCCCTGCGATAC	This study
PGN_0449	GGACTGCTACTGGCTTTCTT/AGCAGACAGATTCCCGAAAG	This study
*luxS* PGN_1474	GCTGCAACTTATCTGCGTAATC/GTAATTGCCTCGCATCAGAAAG	This study
PGN_1085	GCACCTACTCTCTTTCTCCATAC/GCTCCAGATTACAGGTGTCTAC	This study
**EMSA probes**		
*PGN_0753*	CATCTGGATAGCCCTGTTTG/TAAGTGATTGAAAGACTTCG	This study
*hmuY*	TAGATGATTTTCCTTGTCATGCCATAGC/TCTGCGAGATACTGTTTGCTGACAAT	This study
*ragA*	GGATAATAGGATTAGTCT/TAGCGTCATTCTTTTCAT	This study
*rgpA*	CATTTTGATGAAATTAGAA/TGCAAACCCAATATGAGGCC	This study
*kgp*	ACTTTAAAACAATTTATGGTC/CCCTTGTCGCTTATATTGAAA	This study
*hmuS*	CGTATCCGGGTTATACGATCT/CGTGGCGAATTATATTTCTG	This study
*bcp*	CCAACAATACCAATGAGG/AATAGTAAATGCAACACG	This study
*htrA*	ATCCTCGTGACAGCAAGCGTC/TCGAGTAGGGGGAGCGAGC	This study
*nqrA*	GACACAGAATTATTATTC/ACTCTGCACAGGATGGGA	[8]
**Strains and plasmids**		
**Name**	**Description or Genotype**	**Reference**
*P. gingivalis*	ATCC33277(type strain)	
*P. gingivalis*	W83	[37]
TR719	ATCC33277 pT-COW::PG0719	This study
*E. coli* DH5α	*fhuA2 lac(del)U169 phoA glnV44 Φ80’ lacZ(del)M15 gyrA96 recA1* *relA1 endA1 thi-1 hsdR17*	Invitrogen
*E. coli* BL21 (DE3)	*F^−^ dcm ompT hsdS*(r_B_^−^m_B_^−^) *gal (DE3)*	Invitrogen
*E. coli S17*		[5]
pT-COW	Amp^R^ and Tc^ R^ in *E. coli*; Tc^ R^ in *P. gingivalis;* Mob^+^ Rep^+^	[4]
pET-22b^+^	Amp^R^; *pelB; lacI*; T7 promoter; C-terminal his tag	Novagen
pT-0719	pT-COW::PG0719	This study
pET-0753	pET-22b::PGN_0753	This study

Transconjugate strain (TR719) was obtained by *Escherichia coli-P. gingivalis* conjugation. Briefly, the HK (PG0719) from strain W83 was cloned into shuttle/expression vector pT-COW (kindly provided by N. Shoemaker, University of Illinois) [4] by PCR using primers with AvaI and BamHI adapters (Table 1). The resulting plasmid (pT719) was first transformed into *E.coli* S17-1 [5] with selection for ampicillin resistant transconjugants on Luria-Bertani plates (100 µg/ml ampicillin). For filter mating, a culture of plasmid donor strain *E.coli* S17-pT719 was grown to OD_550 nm_ 0.25 and recipient strain ATCC 33277 was grown anaerobically for 48 h on BAP. Donor and recipient were mixed (1∶3 ratio, respectively) centrifuged, and resuspended in 0.5 ml TSB. The suspension was spread on a sterile HAWP047 S0 membrane filter (Millipore) and placed on BAP. A 5 h aerobic incubation (37°C) was followed by overnight anaerobic incubation. Bacteria were harvested in 6 ml TSB, concentrated to 1 ml by centrifugation, and 0.1 ml aliquots were spread on BAP containing tetracycline (3 µg/ml) to select for pT-719 containing *P. gingivalis* colonies, and gentamicin (200 µg/ml) to counterselect the *E. coli* donor. Transconjugants were obtained after 7 days anaerobic incubation, purified, and maintained on BAP containing tetracycline (1.5 µg/ml).

### Purification of PG0720 and PGN_0753 Recombinant Proteins

Recombinant protein PG0720 (the RR from W83) was constructed as a glutathione-S-transferase fusion protein as described previously for FimR [6]. This was used for production of rabbit anti-PG0720 antibody (Covance).

The PGN_0753 (ATCC 33277) and PG0720 ORFs were PCR-amplified using primers listed in Table 1. Each product was cloned into pGEMT-Easy (Promega) for sequencing. After digestion with NdeI and NcoI, the insert was cloned into the NdeI-NcoI site of vector pET-22b^+^ (Novagen) for expression as a His_6_-tagged fusion proteins in *E.coli* BL21DE3 (Novagen). For recombinant protein production, 1 L of insert-containing *E. coli* BL21DE3 was grown to OD_550 nm_ 0.8, then induced with 1 mM IPTG. Cells were disrupted by sonication, recombinant protein was purified by affinity chromatography on Ni^2+^ nitriloacetic (NTA) agarose (Qiagen), and concentrated on Amicon Ultra-4 10,000 NMWL columns (Millipore). Protein concentrations were determined by Bio-Rad protein assay using bovine gamma globulin as a standard. The purity of the recombinant proteins was examined by Western blot using anti-His tag or anti-PG0720 primary- antibodies and horse radish peroxidase-conjugated goat anti-rabbit secondary antibodies.

### *In vitro* and *in vivo* PG0720 Protein*-* DNA Binding Assays

A protocol based on that of Dietz et al., [7], [8] was used to isolate PG0720 protein -DNA complexes that formed *in vitro.* Protein- DNA complexes that formed *in vivo* were identified by ChIP-on-chip under conditions described previously [8]. Anti-PG0720 specific antibodies were purified using an immunoblotting protocol [8], [9].

### Western Blot

*P. gingivalis* strains W83, ATCC 33277 and TR719 strains were grown to OD_550 nm_ 0.5 in TSB (10 ml). Cells were resuspended in 2.0 ml lysis buffer (50 mM Tris-HCl, pH 8.0, 1 mM EDTA, 0.2 mM TLCK), sonicated, and centrifuged (10,000 rpm) to sediment unbroken cells and debris. Equal amounts of protein were mixed with equal volumes of Laemmli sample buffer, denatured by boiling, and loaded onto precast 4 to 20% polyacrylamide gels (Bio-Rad). After SDS-PAGE, fractionated proteins were electro-transferred to nitrocellulose membranes (Bio-Rad) and incubated with primary antibody (rabbit anti-PG0720) for 1 h, and horseradish peroxide-linked goat anti-rabbit secondary antibody (Amersham) for 45 min. Signals were detected with the ECL Western-blot detection kit (Amersham).

### Isolation of RNA from *P.gingivalis* Strains and QRT-PCR

Strain W83 was grown on BAP, and ATCC 33277/pTCOW (empty vector control) and TR719 on BAP containing tetracycline, for 48 h at 37°C under anaerobic conditions. From BAP, strains were cultured in TSB containing 2 µg/ml hemin and 1 µg/ml menadione, and harvested at mid- logarithmic growth phase (OD_550 nm_ 0.5). Total RNA was extracted using the *mir*Vana RNA Isolation Kit (Ambion) according to the manufacturer’s instructions. Contaminating genomic DNA was removed by digestion with Turbo RNase-free DNase I (Ambion) followed by sodium acetate precipitation. DNA contamination was assessed by PCR amplification of using 16S rRNA primers (Table 1), and agarose gel electrophoresis. RNA concentration, purity and quality was determined using NanoDrop N-1000 spectrophotometer (NanoDrop Technologies) and agarose gel electrophoresis.

An aliquot of RNA (1 µg) was reverse transcribed to cDNA with random hexamer primers and RevertAid™M-MuLV Reverse (Fermentas). Real-time PCR was carried out with an iCycler (Bio-Rad). Primers for specific genes (Table 1) were designed with Integrated DNA Technologies Bio Tools (http://www.idtdna.com/scitools/scitools.aspx). Optimization of PCR conditions for each specific primer pair (from Integrated DNA Technologies) was carried out with iQ SYBR green Supermix (Bio-Rad) to detect double-stranded DNA products. The expression of each gene was related to that of the 16S rRNA gene which was used as an internal reference. All reactions were carried out in triplicate. The real-time cycling conditions were: 95°C for 7 min for the initial activation step, 50 cycles each of denaturing at 95°C for 10 s, and annealing-extension at 57°C for 15 s. To confirm that a single PCR product was amplified, melting curve analysis was performed with the following conditions: 95°C for 1 min, 55°C for 1 min, and 55 to 95°C with a heating rate of 0.5°C per 10 s. In addition, amplicons were fractioned on 2% agarose gels to confirm the predicted sizes. Fold-changes in gene expression between ATCC 33277 and TR719 were calculated by the Pfaffl equation, in which the expression ratio is represented as: (Etarget)ΔCttarget(control–experimental)/(E ref)ΔCt ref(control – experimental) [10]. The equation normalizes the expression of the gene of interest (target) and subtracts the expression of the 16S rRNA gene based on PCR efficiency (E) and threshold cycle (Ct), i.e., the cycle number at which exponential fluorescence is detectable. PCR efficiency is obtained from the equation 10^−1/slope^ and is a reliable factor for estimating the quality of the PCR product generated during exponential phase amplification of each gene in a template dilution series. The slope was automatically calculated by the iCycler from the logarithmic plot of the cycle number derived from 10-fold dilutions of a pool of cDNA samples. Theoretically, a slope of −3.3 indicates that the PCR efficiency is 100% or two-fold amplification per cycle.

### Comparative Transcription Profiling

For microarray analysis, an aliquot of RNA (15 µg) for ATCC 33277 parent and TR719 strains was reversed transcribed for 16 h at 42°C with random hexamers, Superscript III (Invitrogen), and 25 mM dNTP/5-(3-aminoallyl)-UTP (Fermentas) mix. After synthesis, unincorporated aa-dUTP and free amines were removed using Qiagen PCR purification columns with phosphate buffers (wash buffer: 5 mM KPO_4_ pH 8.0, 80% EtOH; elution buffer: 4 mM KPO_4_ pH 8.5) instead of the supplied buffers. The aminoallyl-labeled cDNA was subsequently labeled with either Cy3 or Cy5 for 16 h. Unbound dye was removed using the Qiagen PCR purification kit according to manufacturer’s instructions prior to quantitation using a NanoDrop N-1000 spectrophotometer (NanoDrop Technologies). The samples were read at 260 nm for cDNA concentration and either 650 nm for Cy5 incorporation or 550 nm for Cy3 incorporation. Labeled cDNA samples were detected by hybridization to *P. gingivalis* microarrays obtained from the J. Craig Venter Institute, as described previously [9]. The microarray probes were derived from annotated open reading frames (ORFs) from strain W83. Sequences of those which showed hybridization with ATCC 33277 cDNA were used to interrogate the 33277 genome and assigned the number of the gene with the highest homology. The microarray data are deposited in the Bioinformatics Resource for Oral Pathogens database and can be accessed using URL: http://www.brop.org/idn:13736458448418.

### Chromatin Immunoprecipitation and Library Construction for Illumina HiSeq Sequencing

TSB broth without hemin or supplemented with 0.001 µg/ml or 2 µg/ml hemin cultures of transconjugant TR719 were grown to OD_550 nm_ 0.5 followed by the addition of 1% formaldehyde to cross-link DNA-protein complexes that formed *in vivo*. Isolation of DNA-protein complexes and chromatin immunoprecipitation was carried out as previously described using anti-PG0720 antibody and anti-IgG antibody as a negative control [6]. DNA-protein complexes were fragmented to 0.3 kb–1 kb by sonication (Branson sonifier) and used as the input fraction for immunoprecipitation with purified anti-PG0720 antibody or anti-IgG antibody. After treatment with proteinase K, DNA was treated with the Qiagen PCR purification kit according to manufacturer’s protocol. Using the NEBNext ChIP-Seq Library Prep Reagent Set for Illumina (New England Biolabs), samples were prepared for sequencing on an Illumina HiSeq according to manufacturer’s protocol with some modifications. Starting DNA (40 ng) was end repaired, dA-tailed, and ligated to NEBNext adaptors (0.75 µM) using manufacturer supplied buffers and enzymes. The libraries were run on a 2% agarose gel and 0.25 kb–0.5 kb fragments were selected for NEBNext Multiplex Oligos for Illumina (New England Biolabs) were used to PCR amplify the gel purified fragments. PCR products were cleaned using a Qiagen PCR purification kit.

Sequencing was performed using an Illumina HiSeq2000 platform at the Tufts University Core Facility at Tufts Medical Center. ChIP-seq reads were mapped to the *P. gingivalis* ATCC 33277 genome using Galaxy software (https://main.g2.bx.psu.edu/) [11], [12], [13]. The three libraries for each condition were pooled for ChIP-seq and compared to the IgG reference library set.

### Bioinformatic Analyses

ChIP-seq data were mapped to the ATCC 33277 genome using Galaxy software. All four libraries were analyzed with GenomeView (genomeview.org) [14] to identify sequences enriched by immunoprecipitation with anti-RR antibody. Regions with enriched hits for IgG binding were excluded from the search as false positives. Enriched sequences in the libraries of cultures grown under the three hemin conditions were analyzed to determine if the regions were upstream from a gene by no more than 400 bp and sequenced in the direction of its start or directed outward from the start and within the first 75–100 bp of the gene. These criteria allowed for coverage of putative promoter regions. Enriched regions that were identified solely within genes were excluded from this study.

### Electrophoretic Mobility Shift Assays (EMSA)

DNA fragments (200–300 bp) from the 5′ untranslated regions of genes of interest were amplified by PCR from *P.gingivalis* ATCC 33277 chromosomal DNA using *Pfx* polymerase (Invitrogen) using primers used listed in Table 1. The promoter of PG2182 (*nqrA*) from strain W83, the target of RR RprY [8] was used as a negative control. DNA fragments were labeled with the DIG Gel Shift Kit (Roche) according to the manufacturer’s instructions. His_6_-PGN_0753 was induced and purified as described above. In each EMSA reaction, DNA (0.80 pmol/µl) was incubated with 3.5, 40, and 112 pmole PGN_0753 protein in 15 µl total volume binding buffer (50 mM Tris HCl, pH 8.0; 750 mM KCl; 2.5 mM EDTA; 0.5% Triton-X; 2.5% glycerol; 1.0 mM acetyl phosphate, 1 mM DTT). After incubation for 20 min at room temperature, the reactions were mixed with Hi-Density TBE Sample Buffer (Invitrogen), loaded onto native 6%- polyacrylamide precast gels (0.5×TBE, 6% polyacrylamide, 2.5% glycerol) and run at 4°C. DNA-protein complexes were electro-transferred to positively charged nylon membranes (GE Health Care) and incubated with anti-digoxigenin antibody (Roche). Detection with CDP-star was carried out according to the manufacturer’s instructions (Roche).

## Results

### Strain ATCC 33277 is Defective in PGN_0752 Histidine Kinase

**Figure 1:**
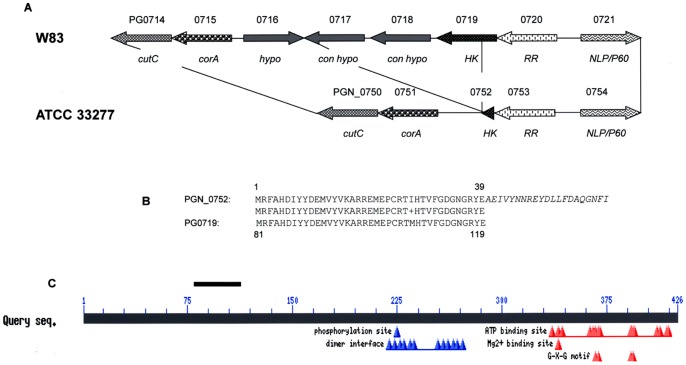
The PG0719-0720 TCS region in strain W83 and the homologous region in ATCC 33277. A. Alignment of loci. Hypo, hypothetical protein; con hypo, conserved hypothetical; HK, histidine kinase; RR, response regulator. B. Protein sequence homology between HKs PG0719 (W83) and PGN_0752 (ATCC 33277). The latter is shown as a chimeric protein containing the italicized C-terminal sequence of the PG0717 homologue. C. Functional domains within PG0719 from strain W83. The bar between amino acids 75 and 150 depicts the homology remaining between PGN_0752 and PG0719.

Our attempts to construct RR or HK mutants in TCS PGN_0752-0753 of strain ATCC 33277 were unsuccessful. Furthermore, we were unable to isolate similar mutants in homologues PG0719-0720 from strain W83, and provisionally concluded that this TCS may be essential. Recently, we used a Mariner-based transposon mutagenesis system to generate mutant libraries in W83 and ATCC 33277 backgrounds [15]. Neither of these libraries contained mutants within the coding region of HK PG0719 or PGN_0752 nor the N-terminal portion of RR PG0720 and PGN_0753 which contains the RR receiver domain, signifying that these genes encoded essential functions. In a previous study we noted that the genomic region surrounding the PG0719-0720 TCS was highly divergent between strains W83 and ATCC 33277 [16]. Following publication of the genome sequence of ATCC 33277 [17] a close examination of this region revealed a 2.529 kbp deletion in ATCC 33277 at the locus homologous to TCS PG0719-720 of W83 (Fig. 1A). As annotated by Naito et al. [17], RR PGN_0753 from ATCC 33277 is 19 amino acids longer than the W83 homologue due to a mutated stop codon and read through into the residual fragment of the HK gene (PGN_0752) until the next stop codon. The 39 amino acids of the HK internal coding sequence resulted from a return to translation up to the start of the deletion (Fig. 1B and C). The deletion removed the rest of the PGN_0752 HK gene including phosphorylation and DNA binding sites and also PG0718, a conserved hypothetical of variable size according to different annotation sources (Bioinformatics Resource for Oral Pathogens, (http://genome.brop.org). In addition, most of the PG0717 (W83) homologue was deleted except for the sequence encoding the 30 C-terminal amino acids which is fused to the 38 amino acid fragment of HK PGN_0752 and shown as the italicized sequence in Fig. 1B.

**Figure 2:**
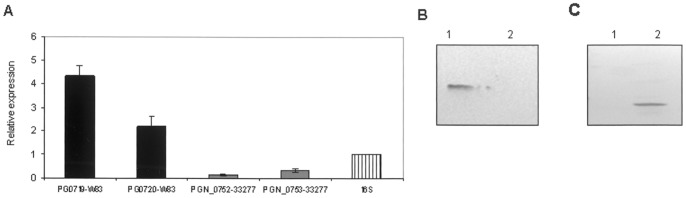
Expression of the TCS in strains W83 and ATCC 33277. A. Quantitative RT-PCR of the HK and RR genes. Results were obtained from five independent cultures of strains W83 and ATCC 33277 grown to OD550 nm 0.5 using 1 mg RNA from each sample. B. Western blot of RR production in strain W83 (lane 1) and ATC C33277 (lane 2). C. Western blot of PGN_0753 response regulator production in ATCC 33277 parent (lane1) and transconjugate TR719 (lane 2). Each lane contains 10 µg of total protein. Blots were probed with rabbit anti-PG0720 primary- and HRP-conjugated goat anti-rabbit secondary antibodies.

To confirm that the HaeRS TCS was not functional in ATCC 33277 we compared expression of the HK and RR transcripts with those from W83 by QRT-PCR and RR protein production by Western. Clearly, in strain ATCC 33277 expression of the HK transcript was barely detectable and that of the RR transcript was approximately five-fold less than in W83 (Fig. 2A). These data were confirmed by Western blot where RR production by ATCC 33277 could not be detected (Fig. 2B). We constructed a transconjugate strain (TR719) of ATCC 33277 that carried the functional HK (PG0719) from W83 on pT-COW and tested whether production of RR PGN_0753 was restored. By Western (Fig. 2 C lane 2), we observed that the presence of functional HK PG0719 (W83) restores expression of RR PGN_0753 in the transconjugant.

### Addition of HK PG0719 from Strain W83 Restores the Growth Defect of ATCC 33277

To prepare for identification of the PGN_0753 regulon by ChIP-seq, preliminary experiments were carried out with RR PG0720 from W83 to determine conditions for immunoprecipitation of DNA-RR complexes that formed *in vivo*, as described previously [6], [8]. The genomic DNA fragments obtained from these initial experiments were identified by hybridization to microarrays and were enriched for promoters of genes potentially involved hemin transport, e.g. *htrA* (PG0648 in W83; PGN_0687 in ATCC 33277), *ihtA* (PG0668; PGN_0704) and *hmu*Y (PG1551; PGN_0558) a TonB-dependent receptor (data not shown)**.** These data suggested that the PG0719-0720/PGN_0752-0753 TCS may be associated with hemin/iron transport, and that growth of ATCC 33277, a naturally occurring mutant in the TCS, may be compromised under iron and/or hemin-restrictive conditions.

**Figure 3:**
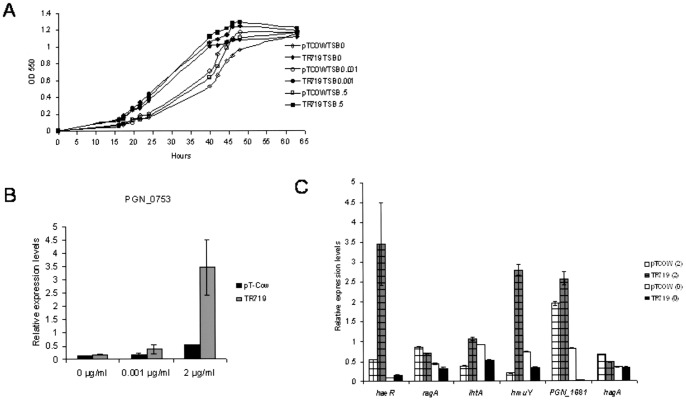
The functional HK from strain W83 restores growth defects of ATCC 33277. A. Growth of strains under hemin-deplete and replete conditions (0, 0.001, and 0.5 µg/ml, respectively: n = 3). B. Expression of RR PGN_0753 in ATCC 33277/pTCOW and TR719 in hemin-deplete, -limited, and -replete conditions measured by QRT-PCR. C. Expression of genes involved in iron/hemin transport in ATCC 33277/pTCOW and transconjugant TR719 grown under hemin- depleted and replete conditions. Data were obtained by QRT-PCR. PGN_1681: ATP-transporter ATP-binding protein.

We compared the ability of strains ATCC 33277 and TR719 to grow under hemin-replete and -limited conditions. Strains were grown anaerobically for 48 h on trypticase soy agar plates (TSA) containing 2 µg/ml hemin and sheep blood (5.0%), washed twice with TBS without hemin then resuspended in TSB with hemin at 2.0, 0.001, and 0.0 µg/ml. Under all three conditions the growth of TR719 during the lag and exponential growth phases was greater than that of the ATCC 33277 parent (Fig. 3A). Interestingly, the parent grew better in very low hemin (0.001 µg/ml) than in the other conditions. By QRT-PCR we determined the presence or absence of PGN_0753 regulator (*haeR*) in the cell extracts from the same cultures and, as predicted based on previous QRT-PCR analysis, the parent strain produced very little HaeR, while strain TR719 strain produced an abundance of HaeR (Fig. 3B).

### Differential Gene Expression in Transconjugant and Parent Strains Induced by Hemin

**Figure 4:**
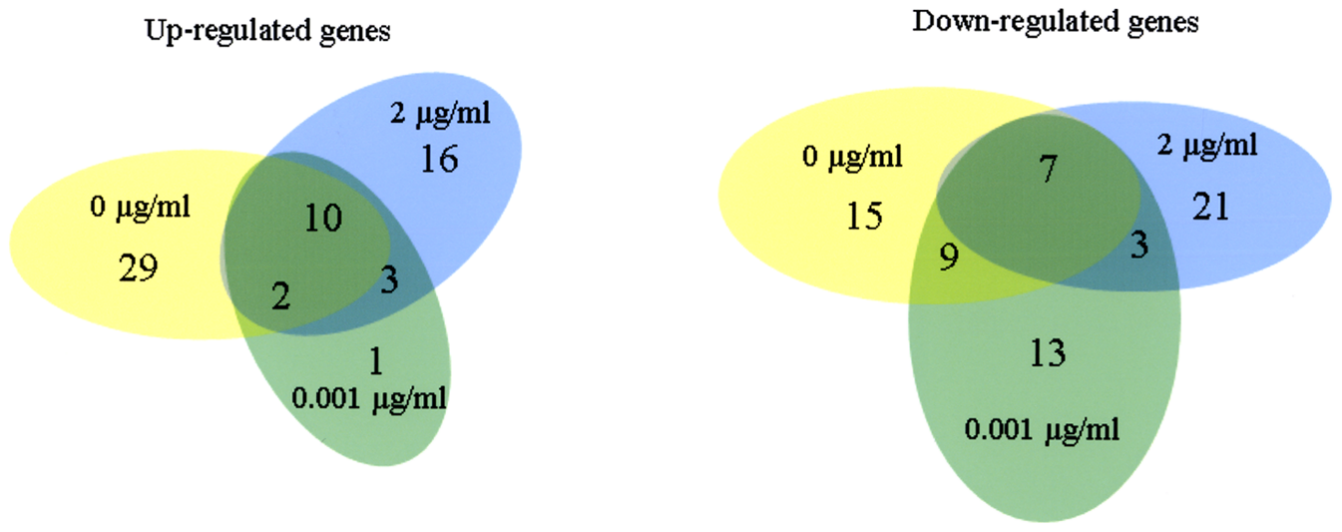
Venn diagram depicting numbers of genes up- or -down regulated in ATCC 33277 during growth in hemin- depleted, -limited and replete media. Gene expression was quantified by microarray and fold changes were calculated by dividing values for transconjugant TR719 by those for ATCC 33277 containing pTCOW empty vector. For up-regulated genes the cutoff was at least a two-fold increase and for down regulated a decrease of at least 0.62.

We compared expression profiles of the parent ATCC 33277 containing pT-COW empty vector and transconjugant strain TR719 under three growth conditions: TSB with 0, 0.001, and 2.0 µg/ml hemin. As shown in Fig. 4, in limited hemin (0.001 µg/ml), a total of 16 genes were up-regulated (>2-fold increase) in TR719 compared to the parent and 32 genes were down-regulated (<0.61-fold). Under standard growth conditions (2.0 µg/ml hemin), 29 genes were up-regulated at least 2-fold in TR719 compared to the parent, while 31 genes were down-regulated. In hemin depleted conditions (0.0 µg/ml hemin), 41 genes were up-regulated (>2 fold increase) in TR719 compared to the parent and 32 were down-regulated.

**Figure 5:**
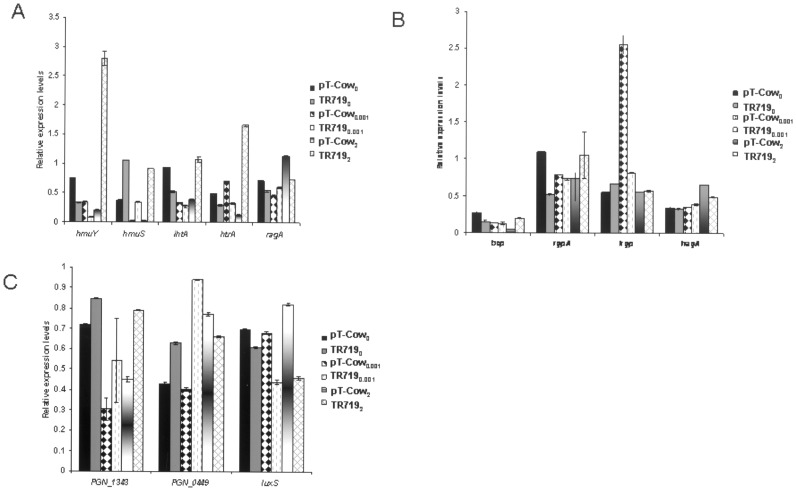
Validation of microarray and ChIP-seq targets by QRT-PCR. QRT-PCR data were obtained from four independent cultures of ATCC 33277/pTCOW and TR719 per hemin condition grown to OD550 nm of 0.5. One mg of RNA was used from each sample. A. Expression levels of major iron transport genes. B. Expression levels of iron acquisition genes. C. Expression levels of genes consistently up- or down-regulated in the microarrays.

In all three conditions two putative operons were up-regulated 2-to 7-fold in the transconjugate strain (Table 2). The first comprised PGN_1343 through to PGN_1349 and is predicted to encode an ABC transporter, a one component protein, a putative TonB-dependent receptor and a lipoprotein. The second up-regulated putative operon comprised PGN_0449 through to PGN_0444 which encodes putative ABC transporter and outer membrane efflux proteins. Relative expression levels of PGN_1343 and PGN_0449 were analyzed by QRT-PCR (Fig. 5). PGN_1343 expression was increased 30% when hemin was limited. A similar trend was observed for hemin-depleted for PGN_1343 (18% increase) and PGN_0449 (45% increase), as well as for hemin-replete for PGN_1343 (75% increase). However, there was a 15% decrease in expression of PGN_0449 in the hemin-replete condition by QRT-PCR.

**Table 2:** Genes upregulated in transconjant TR719 compared to parent ATCC 33277 grown in TSB with variable hemin.

Role Category	Locus	Known or predicted function	Fold change (TR719 vATCC33277/pTCOW)		
			Hemin µg/ml		
			0	0.001	2
Transport	PGN_0444	Outer membrane efflux protein	6.56	3.88	3.12
	PGN_0445	RND family efflux transport MFP subunit	7.81	3.89	3.16
	PGN_0446	ABC transporter; permease protein, putative	5.37	3.11	2.90
	PGN_0447	ABC transporter; permease protein, putative	5.42	2.96	2.55
	PGN_0448	ABC transporter; ATP-binding protein	3.95	2.62	2.50
	PGN_1343	ABC transporter; ATP-binding protein	8.26	5.47	5.87
	PGN_1347	Putative TonB-dependent receptorexported protein	8.47	4.71	9.18
Transcription/Translation	PGN_1415	Histone-like family DNA-binding protein	2.66	2.23	1.75
Enzymes/Metabolism	PGN_1047	Hydroxylamine reductase	0.86	2.96	2.23
	PGN_1349	Prolyl oligopeptidase family protein	6.20	3.40	3.88
Lipoprotein	PGN_1348	Putative lipoprotein	10.08	4.27	5.97
Signaling	PGN_1346	GntR family transcriptional regulator	10.70	4.41	8.97
Hypothetical	PGN_0449	Unknown; possible IM protein	2.16	2.17	1.94
	PGN_1344	Unknown; possible IM protein	n/a	6.34	10.76
	PGN_1345	Unknown; IM protein	11.17	5.63	8.49

Nine genes were down-regulated in TR719 in all three conditions, and two of the genes encode hypothetical proteins (Table 3). One down-regulated gene of interest is PGN_0460 which encodes a histone-like family DNA-binding protein. A second is PGN_1474 encoding S-ribosylhomocysteinase, a member of the LuxS superfamily. We confirmed by QRT-PCR that *luxS* expression is decreased between 13% and 45% under all hemin conditions (Fig. 5). Under hemin-depleted and -limited conditions several important genes involved in heme/iron acquisition are down-regulated, e.g. *hagA* (PGN_1733) and *rgpA* (PGN_1970). As demonstrated by QRT-PCR, under all hemin conditions there was either decreased or equal expression of *hagA*. Since strain ATCC is functionally mutant in the HaeSR TCS, we reasoned that the differentially regulated genes described above were potential components of its regulon.

**Table 3:** Genes downregulated in transconjugant TR719 compared to parent ATCC 33277 grown in TSB with variable hemin.

Role Category	Locus	Known or predicted function	Fold-change (TR719 v ATCC33277/pTCOW)		
			Hemin µg/ml		
			0	0.001	2
Iron/virulence	PGN_0152	Immunoreactive 61 kDa antigen PG91	1.46	0.55	0.50
	PGN_1058	Bacterioferritin comigratory protein; Bcp	0.65	0.22	0.40
	PGN_1733	Hemagglutinin protein; HagA^1^	0.47	0.24	1.11
	PGN_1970	Hemagglutinin protein; RgpA	0.43	0.49	0.98
Transcription/Translation	PGN_0139	rRNA large subunit methyltransferase^1^	0.32	0.31	0.57
	PGN_0392	Competence/damage inducible protein; CinA^1^	0.29	0.26	0.50
	PGN_0450	ECF subfamily RNA polymerase sigma factor^1^	0.39	0.23	0.36
	PGN_0460	Histone-like family DNA-binding protein^1^	0.18	0.17	0.30
	PGN_0472	DNA topoisomerase IV subunit A	0.51	0.25	1.05
	PGN_1590	50S ribosomal protein L13; RplM^1^	0.32	0.20	0.21
	PGN_1932	CRISPR-associated Csm1 family protein	0.19	0.23	1.36
Enzyme	PGN_1457	Probable alkaline phosphatase	0.24	0.13	0.67
Signaling	PGN_1474	S-ribosylhomocysteinase; LuxS^1^	0.22	0.21	0.45
Hypothetical	PGN_0148	Unknown; possible IM protein; AmsA domain^1^	0.21	0.20	0.51
	PGN_0832	Unknown; gliding motility protein; SprA	n/a	0.18	0.40
	PGN_1125	Unknown; IM protein; NfeD domain	0.18	0.33	1.10
	PGN_1145	Unknown^1^	0.36	0.41	0.53
	PGN_1369	Unknown	0.33	0.31	1.10
	PGN_1392	Unknown	0.60	0.29	n/a

1 Down-regulated in all three hemin conditions.

### Identification of HaeR - DNA Binding Regions by ChIP-seq

Cultures were grown under three hemin concentrations (0.0, 0.001, and 2 µg/ml). Complexes of ATCC 33277 genomic DNA and HaeR that formed *in vivo* were precipitated with antibody to rHaeR cloned from strain W83 (PG0720). The regions of DNA complexed with HaeR were sequenced using an Illumina HiSeq 2000 platform giving directional reads of approximately 20–25 bp that were aligned to the genome of ATCC 33277. The reads for the three conditions were normalized to the IgG control then the genome was scanned for regions enriched in the ChIP sequence pool. Our primary goal was to identify new promoter regions bound by HaeR, therefore, we focused on sequences that were located within the putative promoter region of a gene rather than within genes (Table 4). For inclusion, the reads had to be sequenced in the direction of the start of the gene and no more than 400 bp in front of the gene. Alternatively, the read could be directed outward from the start of the gene and within the first 75–100 bp of the gene. The hits nested within genes are the focus of a future investigation. HaeR binding sites were found in front of 57 genes, and in most cases binding occurred in all three hemin conditions. Seventeen of the genes with HaeR sites were associated with transport, e.g. TonB-dependent receptors and transporters and ABC transporters. The next largest class of genes encoded hypothetical proteins among which were several putative inner and outer membrane proteins with possible roles in transport. Of note was a class of genes significant for roles in iron transport and/or virulence, e.g. gingipains and hemagglutinins.

**Table 4:** HaeR targets identified by ChIP-seq.

Role category	Associated locus	Known or predicted function	Hemin µg/ml*		
			0	0.001	2
Transport	PGN_0006	Na+ driven multidrug efflux pump	xx	x	x
	PGN_0142	Cation efflux protein	x	x	xx
	PGN_0687	Putative iron compound ABC transporter	x		
	PGN_0704	Putative TonB-linked outer membrane receptor	xx	xx	x
	PGN_0721	Putative ABC transporter ATP-binding protein	xx	xxx	x
	PGN_0889	TrkA_C domain containing protein	x	x	x
	PGN_0890	Putative TonB-dependent outer membrane receptor protein	xx	xxx	x
	PGN_1085	Ferrous iron transport protein B	x	xx	xxx
	PGN_1207	Putative transport multidrug efflux protein	x	x	
	PGN_1223	Uracil permease	x	x	x
	PGN_1347	Putative TonB-dependent receptor exported protein	x	x	
	PGN_1387	Putative ABC transporter permease protein			x
	PGN_1432	Probable outer membrane efflux protein	xxx	x	xx
	PGN_1458	Preprotein translocase subunit SecA	x	x	x
	PGN_1518	Putative oligopeptide transporter	xx	xxx	x
	PGN_1830	Putative TonB-dependent receptor	x	xx	
	PGN_1953	TonB-dependent outer membrane receptor			x
Iron/virulence	PGN_1058, *bcp*	Bacterioferritin comigratory protein	xx	x	
	PGN_1308	Probable iron dependent repressor	x		
	PGN_1728, *kgp*	Lysine-specific cysteine proteinase	xx	x	x
	PGN_1733, *hagA*	Hemagglutinin	x		x
	PGN_1904, *hagB*	Hemagglutinin	x	x	x
	PGN_1906, *hagC*	Hemagglutinin	x		
	PGN_1970, *rgpA*	Arginine-specific cysteine proteinase	xx	x	x
Metabolism/biosynthesis	PGN_0318	Precorrin-3B C17-methyltransferase	x	x	x
	PGN_0388	Putative thiol peroxidase	x	x	
	PGN_0429	Putative 4-alpha-glucanotransferase	xx	x	
	Intergenic PGN_0433/PGN_0434	Phosphoglycerate kinase/phosphoenolpyruvate carboxykinase	xx	x	x
	PGN_0457	Methylmalonyl-CoA mutase small subunit	xx	xx	x
	PGN_0556	Putative cobalamin biosynthesis-related protein	x	xx	x
	PGN_0606	Glucosamine-6-phosphate deaminase-like protein	xxx	xx	x
	PGN_1530	2-oxoglutarate ferredoxin oxidoreductase subunit	xx	x	x
Signaling	PGN_0753	Response regulator	x		xx
	PGN_0904	Probable sensor kinase	x	x	
Phage integrase/recombinase	PGN_0385	Putative integrase/recombinase XerD	xx	xx	x
	PGN_0917	Tyrosine type site-specific recombinase	x	xx	x
	PGN_1191	Transposase in ISPg1	x	x	x
	PGN_1727	Transposase in ISPg1	x	x	x
Transcription/translation	PGN_0924	Mobilization protein	x	x	x
	PGN_1631	Putative DNA-binding protein, histone-like family	xx	x	x
	PGN_1660	Possible positive regulator of sigma E	x		
Enzyme	PGN_1349	Dipeptidyl aminopeptidase	x	xx	x
	PGN_1685	Malic enzyme	x	x	x
Hypothetical	Intergenic PGN_0186/PGN_0187	unknown	x	xx	x
	PGN_0291	unknown; possible IM protein	xx	xx	x
	PGN_0304	unknown	x	x	x
	PGN_0607	unknown; peptidase S46 domain	x	xx	x
	PGN_0688	unknown	x	xx	xx
	PGN_0712	unknown	x	xx	x
	PGN_1061	unknown	xx	xx	x
	PGN_1067	unknown	x	xx	x
	PGN_1313	unknown; possible IM protein	x	x	xx
	PGN_1459	unknown	x	xx	x
	PGN_1480	unknown; possible IM protein	x	x	x
	PGN_1535	unknown; possible lipoprotein	x	xx	
	PGN_1557	unknown; possible OM protein	xx	xx	x
	PGN_1719	unknown	x	x	x

* An x under a specific hemin concentration indicates that reads were recorded for the associated gene in that given growth condition. More than one x indicates approximately twice the number of reads in that condition compared to the others.

### Direct Regulation of Genes by HaeR Confirmed by EMSA

**Figure 6:**
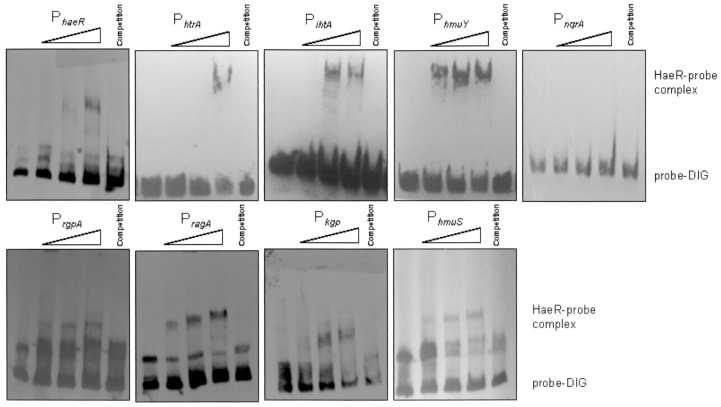
EMSA with RR PGN_0753 and promoters of genes involved in hemin/iron acquisition. The 5′ regulatory regions of *haeR* (PGN_0753), *htrA* (PGN_0687), *ihtA* (PGN_0704), hmuY (PGN_0558), *rgpA* (PGN_1970), *ragA* (PGN_0293), *kgp* (PGN_1728), and *hmuS* (PGN_0558) were PCR-amplified and DIG-labeled. Recombinant PGN_0753-His6 (3.5 pmole, 40 pmole, 112 pmole) was incubated in phosphorylation buffer with labeled targets (0.80 pmol/ml). Specific competitions were performed with 100-fold excess of each unlabeled probe. The promoter of *nqrA* (PG2182), a known target gene of RR RprY, was used as a negative control for PGN_0753 binding.

Electromobility shift assays were used to confirm a role for HaeR in the direct regulation of genes involved in hemin transport, gingipain production, and others revealed by microarray and ChIP-seq (Fig. 6). Promoter sequences of approximately 200–300 bp were generated by PCR from strain ATCC 33277 genomic DNA using primers designed from regions 5′ to each ORF (Table 1). Increasing concentrations of recombinant His_6_-PGN_0753 (3.5, 40, and 112 pmole) were incubated with promoter sequences (0.8 pmole/µl). In each case addition of rPGN_0753 lead to retardation of the electrophoretic mobilities of the putative promoters of *htrA*, *ihtA*, and *hmu*Y; addition of excess probe ablated binding and hence retardation. In addition to *hmu*Y, PGN_0753 also bound and shifted a region 250 bp directly upstream of *hmuS*, the third gene in the *hmu*Y*RSTUV* operon that was identified as a potential target of HaeR by ChIP-seq. PGN_0753 did not bind to the promoter of *nqrA* (PG2182), a target of regulator RprY that was used as a negative control [8]. In addition, we showed that HaeR bound to its own promoter as well as those of *ragA* (PGN_0293) which contains a TonB heme binding motif [18], and also to the promoters of PGN_1728 and PGN_1970, *kgp* and *rgpA*, respectively. Thus, HaeR appears to play a central role in the acquisition and transport of heme by *P. gingivalis.*

In addition, PGN_0753 also binds to putative promoter regions of several genes that encode for signaling proteins, channels, and lipoproteins including PGN_1432, a putative outer membrane efflux protein; PGN_0809, a putative TonB-dependent receptor protein; PGN_1739, a putative lipoprotein; and PGN_1752, a putative ferredoxin 4Fe-4S protein, as well as the promoter region of a one- component protein, PGN_0102, which has, as yet, no known function.

## Discussion

The objective of this study was to define the role of the PGN_0752-PGN_0753 TCS (HaeRS) in the physiology of *P.gingivalis* strain ATCC 33277. When we compared loci of these systems in sequenced strains W83 and ATCC 33277 we noted a deletion in the latter that removed three ORFs upstream from, and most of the HK, PGN_0752 (Fig. 1A). Mariner transposon mutants were not obtained in this HK from either strain indicative of its essentiality [15]. On the other hand, in both strains transposon mutants were obtained in the C-terminal DNA-binding domain of the RR. The regulators PG0720 and PGN_0753 have BLASTP matches (e-values >1×10^−10^) to 16 genes in the Database of Essential Genes (http://tubic.tju.cn/deg/). These matches are exclusively to two-component systems and transcriptional regulators. The RRs of the HaeRS system were similar in both strains although the ATCC 33277 homologue, PGN_0753 was longer by 19 amino acids at the C-terminus as a mutated stop codon lead to read through of a protein fragment encoded in the residual HK DNA sequence (Fig. 1 B). PG0719, the HK from strain W83, has three DEG matches (e-values >1×10^−6^) to the sensory box of the VicK histidine kinase of *Staphlococcus aureus*, the YycG histidine kinase of *Bacillus subtilis*, and the PhoR histidine kinase of *Salmonella. typhimurium*. PGN_0752, in the truncated form present in ATCC 33277 has no match in the DEG.

In the W83 genome, the DNA corresponding to the ATCC 33277 deletion encodes three hypothetical genes (Oralgen annotation, Los Alamos National Laboratory viewed at BROP) and interrogation of the non-redundant nucleotide database (NCBI) showed 100 and 99% identity only to *P. gingivalis* strains W83 and TDC60, respectively. According to the TIGR annotation (shown in Fig. 1A), PG0716–PG0718 are not present, at least in the same genomic region, in strain ATCC 33277. In fact, PG0716 is only annotated as a hypothetical protein-coding sequence in one (Oralgen) of four recognized annotations of the genome, and has neither a Pfam domain nor a DEG match. PG0718, annotated as a “conserved hypothetical” protein has no homology to any ATCC 33277 or DEG genes. Obviously, PG0716 and PG0718 are not essential for growth of ATCC 33277 as no homology was found within the ATCC 33277 genome to these two ORFs. Annotated as a lipoprotein, PG0717 contains four domains of unknown function, Pfam DUF2874; to date, this domain is only found in anaerobic and microaerophilic bacterial species. PG0717 matches to a putatively essential gene in *Bacteroides thetaiotaomicron*. While a PG0717 homologue is not present in the genome of ATCC 33277, one copy of DUF2874 is reconstituted in the chimeric protein comprising the HK PGN_0752 internal fragment and the C-terminal fragment of the PG0717 (Fig. 1B), raising the possibility that essentiality may be functionally complemented by this region. In ATCC 33277 the expression of both genes of the TCS was extremely low, and RR PGN_0753 could not be detected by Western blot, consistent with the transcription data (Fig. 2).

Preliminary assays suggested an association of the TCS with hemin and/or iron transport. A hemin-dependent growth phenotype of the ATCC 33277 parent strain was confirmed by its decreased growth on hemin-replete, -restricted, and -depleted media compared to the transconjugant complemented with the functional HK (PG0719) from strain W83 which partially restored growth of ATCC 33277 under hemin-limiting conditions (Fig. 3A). We used both transcription profiling and ChIP-seq to identify components of the HaeR regulon. Transcription profiling compared gene expression in ATCC 33277 and transconjugant TR719 during growth in hemin-depleted, -restricted, and -replete conditions and identified genes that were directly and indirectly regulated by HaeR. ChIP-seq was carried out with cultures of TR719 incubated under the same hemin conditions in order to identify DNA sequences that are recognized and bound by, and thus directly regulated by HaeR. A subset of genes identified in the microarrays were also identified by ChIP-seq, as were genes previously reported to respond to hemin depletion or excess, e.g. PGN_0687 (*htrA*), PGN_0688 (Ton B receptor), PGN_1728 and PGN_1970, *rgpA* and *kgp*, respectively [19], [20]. Other genes consistently showed increased transcription under all hemin conditions but did not appear in the ChIP-seq sequences suggesting that they are indirectly regulated by HaeR, e.g. gene cluster PGN_0444-0449 that encoded efflux and ABC transporters. Another cluster (PGN_1343-1349) upregulated in all three hemin conditions also encoded ABC and TonB transporter components, however, associated non-coding sequences were detected by ChIP-seq and HaeR binding to promoter regions was established by EMSA.

Primarily, the ChIP-seq data indicated HaeR binding to specific promoter sequences, however, the number of copies of a promoter sequence in the ChIP-seq pool also increased or decreased in response hemin concentration reflecting expression of HaeR targets. For example, the number of pPGN_0753 (*haeR*) sequences in the pool increased from 7 to 68 copies in 0.0 and 2.0 µg/ml hemin, respectively, which we interpreted as the result of an increase in RR activation. Increased activation correlated with increased expression since the QRT-PCR data showed that at 2 µg/ml hemin expression of *haeR* increased 5 to 9- fold relative to the parental level (ATCC 33277 containing pTCOW empty vector), while at 0 µg/ml hemin there is approximately a 2- fold increase in expression of *haeR*, so in this case hemin-activated HaeR appeared to act as a positive regulator. Conversely, expression of gingipain *rgpA* was down-regulated in the transconjugant in response to decreasing hemin, and by ChIP-seq there were more copies of the *rgpA* promoter in the absence of hemin, indicating that hemin-activated PGN_0753 acted as a negative regulator and repressed expression of *rgpA*. Similar results were obtained for the promoters of *kgp*, *hagA*, *hagB* and *hagC* implying that these genes were also induced in the absence of hemin.

In addition to the hemin-responsive pattern observed for the *rgpA* and *haeR* promoters, another was revealed for several promoters by comparing ChIP-seq and QRT-PCR and/or microarray data. The number of ChIP-seq hits for the promoter of PGN_1085 (ferrous iron transport protein B) had a positive correlation with increasing concentrations of hemin (0 µg/ml hemin, 37 hits; 0.001 µg/ml, 76; 2 µg/ml, 161). But by microarray, PGN_1085 was down-regulated in the hemin-replete condition, and no fold- change under limiting hemin, suggesting that HaeR represses expression of PGN_1085 as the hemin concentration increases. An inverse correlation was seen with the promoter of *bcp* (PGN_1058, a putative bacterioferritin comigratory protein) since by ChIP-seq there were fewer hits with increasing hemin concentration, and *bcp* was more down-regulated in hemin-limited and –replete conditions than in the hemin-depleted condition, suggesting that when HaeR does not bind to the promoter transcription is reduced. Based on the data from microarray and ChIP-seq, we conclude that HaeR can act as a repressor and activator depending on both the promoter region and hemin concentration.

ChIP-seq data identified three classes of promoters based on numbers of their sequences in the sequencing pool at specific hemin concentrations. In the first class, the number of bound HaeR sequences increased as the hemin concentration increased, which occurred clearly with six promoter regions. The second class contained promoters that had decreased HaeR –binding with increasing hemin concentration as observed with fifteen promoters. The third category, observed with forty promoters, did not follow any obvious hemin-dependent binding trends.

Among the targets of RR PGN_0753 identified by ChIP-seq were 5′ untranslated sequences upstream of PGN_0704 (*ihtA*), PGN_0687 (*htrA*), and PGN_0556 (*hmuS*). RR binding to these sequences was confirmed by EMSA, therefore we conclude that the three loci, consistent with previously established roles in hemin transport [21], [22], [23] are directly regulated by PGN_0753. Furthermore, the ChIP-seq data indicated greater binding of HaeR to these promoters under hemin- depleted or -limited conditions, i.e. larger numbers of these sequences were in the ChIP-seq pool. The first locus, *ihtABCDE*, encodes a TonB-linked receptor and an ABC transporter cassette. IhtA (PGN_0704) is a TonB-linked receptor recently determined to be a BtuB homolog, an outer membrane cobalamin receptor protein [24]. A coenzyme B12 riboswitch is located upstream of PGN_0704 [24]. Because acquisition and transport of iron into bacteria is essential for their growth the regulation of these processes is complex. The major uptake mechanism is via TonB-dependent transporters, however, their regulation depends not only on regulators such as on two-component systems but also ECFs and small RNAs, including riboswitches [25]. As found in the present study, the promoters of several HaeR- regulated genes also contained regulatory elements at the RNA level, e.g. PGN_0704 (riboswitch), PGN_0556 (stem-loop structure), and PGN_1932 (Clustered Regularly Interspaced Short Palindromic Repeat [CRISPR] element). CRISPR elements are found in most bacteria and have mostly unknown function, however, are believed to offer resistance to bacteriophage and potentially silence DNA [26]. Whether HaeR interacts with these or other regulatory elements will be the focus of future work. The second gene in the locus (*ihtB*, PGN_0705) encodes an outer membrane chelatase that removes iron from hemin for TonB receptor-mediated transport [22]. The second locus, *htrABCD* (PGN_0687-PGN_0684) encodes an ABC transporter and a TonB-linked receptor (*tlr,* PGN_0683). A comparison of the proteome and transcriptome of *P. gingivalis* strain W50 in response to hemin excess or limitation during growth in continuous culture identified upregulation of the *htr* and *hmu* loci upon hemin limitation [20]. Curiously, none of the *iht*, *htr* or *hmu* genes were found in our transcription analyses, possibly because of transcript instability or lack of expression under our experimental conditions.

The third gene cluster identified by ChIP-seq, *hmu*Y*RSTUV*, is the best characterized hemin transport system in *P. gingivalis*. Lewis et al., [21] showed that in strain W83, *hmu*Y, the first gene in the operon, was highly transcribed even in the presence of hemin, and reported differential regulation within the cluster with higher levels of transcription for promoter proximal than promoter distal genes. In the present study, the DNA sequence 5′ to the first gene *hmu*Y was not enriched for PGN_0753 binding, rather the region upstream of the third gene, *hmuS*, was a target of PGN_0753 according to the ChIP-seq data, and binding was confirmed with EMSA. PGN_0556, *hmuS*, is predicted to encode a cobalamin biosynthesis-related protein and contains a stem-loop structure at the 5′ end of the gene which may allow for differential expression of the operon during translation [27]. The region bound by PGN_0753 in the *hmu* locus may function as an activator during transcription, possibly by eliminating the stem-loop structure and permitting RNA polymerase read through and increased expression of the genes downstream of *hmuR*. As demonstrated by the QRT-PCR data, the presence of HK PG0719 in ATCC 33277 increased the relative expression of *hmuS* approximately 15- fold (hemin limited) and 39- fold (hemin replete) above the expression observed in the parent strain.

In addition to the *iht, hmu,* and *htr* transport systems for heme/iron, HaeR binding to the promoter regions of several other genes was also detected by ChIP-seq, e.g. TonB-dependent receptors, putative ABC transporters, and efflux-associated proteins. Of note are PGN_1085 (encodes ferrous iron transport protein B), and PGN_0890 and PGN_1830 that encode a TonB-dependent receptor with CirA domains that mainly transport iron [28]. While TonB-dependent receptors transport heme across the outer membrane, and ABC transporters carry heme across the periplasm and inner membrane [29], cysteine proteinases comprise an important class of proteins that play a role in heme acquisition by releasing and binding heme from hemoglobin and other host proteins. The ChIP-seq data show that the promoter regions of RgpA and Kgp, were enriched under heme-deficient and -limiting conditions. The first indication of Kgp involvement in heme accumulation came from a key genetic study showing that *kgp* mutant colonies did not present the normal black-pigmentation phenotype due to heme adsorption at the cell surface [30]. The mutants also produced less of a protein previously identified as a peptide component of the adhesin domains of Kgp and RgpA [31]. Newer work showed that both proteinases were responsible for the capture of hemoglobin, its degradation and release and conversion of heme to m-oxo bishaem aggregates [32], [33]. Most recently, gingipain and HmuY activities have been linked together in the release of heme from proteins degraded by gingipains and its capture by HmuY [34].

In summary, our study of the HaeSR two-component system of *P. gingivalis* allows us to conclude that it is essential for growth because of our inability to construct an RR deletion mutant in strains W83 and ATCC 33277, and the lack of transposon insertions in the HK in libraries of either strain. The natural HK mutation in ATCC 33277 compromised growth although the RR was retained and expressed at very low levels which may be sufficient for binding and activation of essential targets, ensuring survival. The HaeR regulon includes a number of iron uptake/acquisition genes, as well as those encoding transporters and metabolic functions. In addition, HaeR can act as an activator and a repressor depending on the target as well as hemin concentration during growth. Our data suggests that the TCS is induced by low concentrations of hemin since the ChIP-seq data indicated increased expression and binding of RR PGN_0753 to promoters in hemin-depleted or -limited conditions. Finally, we demonstrated that the HaeSR regulon includes, and HaeR directly regulates expression of, Kgp and RgpA, multifunctional virulence factors of *P. gingivalis.* Previously, it was demonstrated that GppX, a hybrid sensor kinase-response regulator protein, was associated with the maturation and localization of the gingipains on the cell surface, but did not affect transcription [35]. Our study has filled a knowledge gap in the complex and essential pathways for iron/heme utilization by *P. gingivalis*.
